# Supplementary material for: International consultation on incontinence questionnaire – Urinary incontinence short form ICIQ-UI SF: Validation of its use in a Danish speaking population of municipal employees
Source: PLoS One. 2022 Apr 6;17(4):e0266479. doi: 10.1371/journal.pone.0266479 (PMC8986014; doi:10.1371/journal.pone.0266479)
Supplement: S1 Table — (DOCX) [file pone.0266479.s001.docx]

**Supporting information**

**Table 1: The questionnaire**

| **Item** | **Domain** | **Statement, Danish** | **Statement, translated to English** |
| --- | --- | --- | --- |
| 1 | Baseline characteristics | Udfyld dags dato | Fill in date of today |
| 2 | Baseline characteristics | Angiv i hvilken kommune du arbejder | Enter your working place municipality |
| 3 | Baseline characteristics | Angiv venligst din jobtitel | Enter your job title |
| 4 | Baseline characteristics | Angiv dit køn | Enter your sex |
| 5 | Baseline characteristics | Angiv din alder | Enter your age |
| 6 | Baseline characteristics | Angiv din højde | Enter your height |
| 7 | Baseline characteristics | Angiv din vægt | Enter your weight |
| 8 | Baseline characteristics | Ryger du? | Do you smoke? |
| 9 | Baseline characteristics | (Hvis ja) Hvor mange år har du røget? | (If yes) How many years have you been smoking? |
| 10 | Baseline characteristics | (Hvis ja) Hvor mange cigaretter ryger du om dagen? | (If yes) How many cigarettes do you smoke a day? |
| 11 | Baseline characteristics | (Hvis nej) Har du røget tidligere? | (If no) Have you smoked before? |
| 12 | Baseline characteristics | Angiv venligst din civilstatus | Please enter your marital status |
| 13 | Baseline characteristics | Hvor mange timer arbejder du i gennemsnit på din arbejdsplads om ugen? | In average, how many hours do you work per week? |
| 14 | Baseline characteristics | Hvad er dit højeste gennemførte uddannelsesniveau? | What is your highest completed level of education? |
| 15 | Baseline characteristics | Hvad er din stillingsbetegnelse? | What is your job title? |
| 16 | Baseline characteristics | Lider du af en eller flere kroniske sygdomme? | Do you suffer from one or more chronic diseases? |
| 17 | Baseline characteristics | (Hvis ja) Angiv venligst hvilken/hvilke kroniske sygdomme | (If yes) Please enter which chronic disease(s) |
| 18 |  | Oplever du eller har du tidligere oplevet at være utæt for urin? | Do you experience or have you previously experienced leakage of urine? |
| **19** | **VersionDUGS ICIQ-UI SF** | **Hvor tit lækker du urin? (sæt kryds i én boks)** | **https://iciq.net *** |
| **20** | **VersionDUGS ICIQ-UI SF** | **Vi vil gerne vide, hvor stor en mængde urin, du tror du lækker. Du skal sætte kryds ud for den rubrik, der svarer til den mængde du oftest lækker (hvad enten du benytter beskyttelse eller ej)?** | **https://iciq.net *** |
| **21** | **VersionDUGS ICIQ-UI SF** | **Hvor meget generer urinlækagen din i din dagligdag** | **https://iciq.net *** |
| **22** | **VersionDUGS ICIQ-UI SF** | **Hvornår lækker du urin? (sæt kryds i alle de bokser, som passer på dig)** | **https://iciq.net *** |
| 23 | Work related QOL | Hvor meget generer det dig? | How much does it bother you? |
| 24 | Work related QOL | Hvor meget påvirker din urininkontinens alt i alt dit arbejde? Vælg mellem 0 (slet ikke) til 10 (betydeligt) | Overall, how much does you urinary incontinence affect your work? |
| 25 | Work related QOL | Hvor meget bekymrer du dig om din urininkontinens i forhold til dit arbejde? Vælg mellem 0 (slet ikke) til 10 (betydeligt) | In relation to your work, how much do you worry about your urinary incontinence  Choose between 0 (not at all) and 10 (significantly) |
| 26 | Work related QOL | Er der ting du ikke kan deltage i på dit arbejde pga. urininkontinens? | Do you experience situations where you are not participating due to urinary incontinence? |
| 27 | Work related QOL | Har du nogensinde sygemeldt dig pga. urininkontinens? | Have you ever reported sick leave due to urinary incontinence? |
| 28 | Work related QOL | Hvor mange gange i løbet af det seneste år, har du været i kontakt med sundhedsvæsnet grundet din urininkontinens? | Through the last few years, how many times have you been in contact with the health care system due to your urinary incontinence? |
| 29 | Work related QOL | Oplever du at din nattesøvn bliver afbrudt pga. din urininkontinens? | Is your night sleep interrupted by your urinary incontinence? |
| 30 | Work related QOL | Jeg har undladt at søge visse jobs på grund af min urininkontinens. | I have avoided to apply for certain jobs due to my urinary incontinence |
| 31 | Work related QOL | Jeg har ikke søgt jobs langt væk fra min bopæl grundet min urininkontinens. | I have not applied for jobs far away from my home due to my urinary incontinence |
| 32 | Work related QOL | Jeg forsøger så vidt muligt at undgå fysisk krævende arbejdsopgaver grundet min urininkontinens. | As much as possible, I try to avoid physically demanding work tasks due to my urinary incontinence |
| 33 | Work related QOL | Jeg forsøger så vidt muligt at undgå store forsamlinger, fx præsentationer og møder, grundet min urininkontinens. | As much as possible, I try to avoid large gatherings, e.g. presentations and meetings, due to my urinary incontinence |
| 34 | Work related QOL | Jeg forsøger så vidt muligt at undgå rejseaktivitet grundet min urininkontinens. | As much as possible, I try to avoid travel activities due to my urinary incontinence |
| 35 | Work related QOL | Skriv den alder du har, når du påtænker at trække dig fra arbejdsmarkedet? | Write the age you have when you intend to retire from the labor market? |
| 36 | Work related QOL | Har din urininkontinens noget at gøre med, hvornår du påtænker at trække dig fra arbejdsmarkedet? | Does your urinary incontinence have anything to do with your plan for retirement? |
| 37 | Work related QOL | Hvor mange gange har du over det seneste år været i kontakt med sundhedsvæsnet grundet andre årsager end inkontinens? | Through the last few years, how many times have you been in contact with the health care system due to other reasons than urinary incontinence? |
| 38 | Work related QOL | Egne kommentarer. Her har du mulighed for at tilføje egne kommentarer til spørgeskemaet. | Own comments. Here you have the opportunity to add your own comments to the questionnaire. |

* The original ICIQ-UI SF in English and all translated versions can be found on ICIQ’s official webpage and all copyrights of these are preserved by ICIQ.
