# Supplementary material for: International consultation on incontinence questionnaire – Urinary incontinence short form ICIQ-UI SF: Validation of its use in a Danish speaking population of municipal employees
Source: PLoS One. 2022 Apr 6;17(4):e0266479. doi: 10.1371/journal.pone.0266479 (PMC8986014; doi:10.1371/journal.pone.0266479)
Supplement: Attachment — Submitted filename: Response to Reviewers.docx [file pone.0266479.s002.docx]

**Peter F.W.M. Rosier, M.D. PhD
Academic Editor
PLOS ONE
Resubmission date: 10.01.2022**

Dear Mr. Rosier,

We thank you as well as the reviewers for your time, your expertise and your useful valuable comments to improve our manuscript.

In accordance with your inquiries, we have:

- Checked and updated our submission in relation to PLOS ONE’s style requirements.
- Provided additional information about consent in the Ethics section and in the online formula.
- Revised the reference list.

In the following, we answer each comment from Reviewer 1 and 2, starting with the major comments and then the minor. However, first we have one major change in the manuscript, that we would like to account for:

In relation to the revision, I recalculated all the statistics of the results, which led to the discovery of a incorrect item which was included in the previous calculation of Cronbach’s alfa. I recalculated the Cronbach’s alfa for the three correct items (included in the ICIQ-score) and discussed the result with my supervisors/co-authors.

Of course, we prefer transparency about our incorrect calculation included in the first manuscript draft and have now corrected the result of Cronbach’s alfa and the relevant paragraphs surrounding the Cronbach’s alfa in the results, discussion and conclusion sections.

We hope that our honesty and scrupulous revision will be well received and appreciated.

**Reviewer 1:**

**#Major comment 1**: Could it be a problem to have two versions of the ICIQ-UI SF in Danish? Did you consider comparing the two versions in a study? Would it be possible to merge the two versions?

*VersionDUGS is widely used in Denmark and especially within the field of gynecology, where it is used for reporting to the Danish database (DugaBase).
The difference between the two versions does not involve the three items used for the ICIQ-score. Therefore, and for practical reasons, we decided only to validate VersionDUGS.
VersionDUGS is validated for women by Clausen et al. and we continued by validating VersionDUGS in a population with both men and women.*

**#Major comment 2**: It would be of interest to the reader know the absolute reliability.

*We have added Standard Error of Measurement to supplement our ICC.*

**Answer to minor comments:**

*- “Gaussian distribution” is changed to “Normal distribution”*

*- “2.3 agreement” has been replaced with just “ICC (agreement)” since the “2.3” information won’t be useful to the readers of the article.*

*- Information is added about recruitment to both the pilot test and the retest.*

**Reviewer 2:**

**#Major comment 1**: I suggest improving discussion with some of the validation studies which have demonstrated higher Cronbach-alpha value.

*We have included validation studies with higher values of Cronbach’s alfa as requested (page 11 line 217).*

**#Major comment 2**: Also, there is need for improvement in English style and overall language.

*We have to our very best tried to improve the overall language and the manuscript was proofread by a medically knowledgeable with English as his mother tongue.*

All changes are marked with yellow in the manuscript with track changes.

We look forward to hearing from you regarding our submission. We will thorughly respond to any further questions or comments that you might have.

Kind regards on behalf of the author group
Lærke Cecilie Grøn Jensen
Medical Student, Faculty of Health, Aarhus University, Denmark
Vennelyst Boulevard 4, 8000 Aarhus C
[laerke-cecilie@hotmail.com](mailto:laerke-cecilie@hotmail.com)
Tel.: +45 30 69 96 74
