# Supplementary material for: International consultation on incontinence questionnaire – Urinary incontinence short form ICIQ-UI SF: Validation of its use in a Danish speaking population of municipal employees
Source: PLoS One. 2022 Apr 6;17(4):e0266479. doi: 10.1371/journal.pone.0266479 (PMC8986014; doi:10.1371/journal.pone.0266479)
Supplement: Attachment — Submitted filename: Response to Reviewers.docx [file pone.0266479.s003.docx]

**Peter F.W.M. Rosier, M.D. PhD
Academic Editor
PLOS ONE
Resubmission date: 17.03.2022**

Dear Mr. Rosier,

We thank you as well as the reviewers for your time, your expertise and your useful valuable comments to improve our manuscript. We are very grateful to the reviewers for their comments on our manuscript.

All changes are marked with yellow in the manuscript with track changes and we hope that these revisions are sufficient to make our manuscript suitable for publication in PLOS ONE.

Here is a point-by-point response to the reviewers’ comments:

**Reviewer 1:**

Thank you for your revised manuscript
As requested, you have provided the equation for the calculation of absolute reliability (Standard Error of Measurement (SEM)) and you also very shortly report the result from your calculation, but you have not explained what it means in relation to the reliability of the ICIQ-VersionDUGS.
It seems that you have calculated a SEM (68% at group level), but you do not tell what 1.57 means to the test-retest reliability of the ICIQ-VersionDUGS. Furthermore, this figure would be 3.1, if you had calculated SEM (95% at group level), which is relevant for research settings (SEM x1.96), and 4.3 at an individual level. The latter representing the smallest real difference (SRD) (SEM x 1.96 x √2).
The implications of the results above should be described and discussed since it is important knowledge for both researchers and clinicians.
In your discussion page 12, line 234 you write:” The intraclass correlation coefficient of 0.73 is moderate, which is confirmed in the calculated standard error of measurement”. Why does the ICC affect SEM and could you have calculated the SEM based on another analysis (ANOVA)?
Line 235: should be a zero.

**Answer to reviewer 1:**

We thank reviewer 1 for the valuable suggestions and we agree that we could improve our use and presentation of SEM. We have therefore been in contact with a Danish expert in questionnaire technique and clinemetrics to ensure that we use the appropriate equations in our validation study.

We were guided to use the following equations to calculate the appropriate SEM to our ICC:

$SEM=SD \sqrt{1-ICC}$	$SD=(SD_{time1}+SD_{time2})/2$.

We include these equations and the calculations in the manuscript to make the results transparent and to enable readers of our manuscript to calculate further on the numbers.

As you suggested, we furthermore calculated the smallest real difference. A comment on the SEM and SRD is added to the discussion (p. 13 line 233) as well as in the conclusion (p. 14 line 257).

**Reviewer 2:** Thank you for the opportunity to re-revise this manuscript. The revised version is fully acceptable for publication in PLOS ONE

**Answer to reviewer 2:**

Thank you very much for this comment. We are happy to read that you find our manuscript acceptable for publication.

We look forward to hearing from you and will happily respond to any further questions or comments you may have.

Kind regards on behalf of the author group
Lærke Cecilie Grøn Jensen
Medical Student, Faculty of Health, Aarhus University, Denmark
Vennelyst Boulevard 4, 8000 Aarhus C
[laerke-cecilie@hotmail.com](mailto:laerke-cecilie@hotmail.com)
Tel.: +45 30 69 96 74
